# Tianwang Buxin Granules Influence the Intestinal Flora in Perimenopausal Insomnia

**DOI:** 10.1155/2021/9979511

**Published:** 2021-11-16

**Authors:** Xiqian Yang, Hesong Xiao, Yi Zeng, Liangliang Huang, Ke Ji, Diyang Deng, Wenyu Yang, Ling Liu

**Affiliations:** ^1^The TCM Clinical Institute, Hubei University of Chinese Medicine, Hubei 430065, China; ^2^The First Clinical Institute, Hubei University of Chinese Medicine, Hubei 430065, China; ^3^Encephalopathy Department, Hubei Provincial Hospital of TCM, Hubei 430061, China

## Abstract

**Methods:**

The subjects included 13 PI patients from the Hubei Provincial Hospital of TCM, Hubei University of TCM, and Wuhan Traditional Chinese Medicine Hospital, and the corresponding noninsomniac spouses of the patients were selected as controls. TWBXG was continuously administered for 4 weeks. The feces of PI patients and their noninsomniac spouses before and after treatment with TWBXG were collected. The intestinal flora composition of each group was detected by metagenomic sequencing, and the efficacy of TWBXG was evaluated by the PSQI scale.

**Results:**

Compared with the control group, the model group showed an increase in the abundance of Roseburia faecis, Ruminococcus, Prevotella copri, Fusicatenibacter saccharivorans, and Blautia obeum, while those of Bacteroides, fecal Bacteroidetes, and Faecalibacterium prausnitzii were decreased. Compared with pretreatment, the PSQI score was significantly reduced (*P* < 0.05), the abundance of Bacteroides, fecal Bacteroidetes, and Faecalibacterium prausnitzii increased, and that of Roseburia faecis, Ruminococcus, Prevotella copri, Fusicatenibacter saccharivorans, and Blautia obeum decreased after treatment. However, there was still a certain gap in the abundance of related flora in the treatment group compared with the control.

**Conclusion:**

PI is associated with disturbances in the intestinal flora and is mainly related to the disorders of Roseburia faecis, Ruminococcus, Prevotella copri, Fusicatenibacter saccharivorans, Blautia obeum, Bacteroides, fecal Bacteroidetes, and Faecalibacterium prausnitzii. TWBXG can effectively treat PI, and its effect may be achieved by regulating the disordered intestinal flora. *Clinical Trials.* The study was registered in the Chinese clinical trial registry and approved by the World Health Organization clinical trial registration platform (Effects of the modified Tianwang Buxin granule and modified Tianwang Buxin decoction pieces on insomnia: a randomized, controlled trial, ChiCTR-IPR-17011549).

## 1. Introduction

Insomnia is a sleep disorder caused by difficulty in falling asleep or maintaining sleep at night. It results in insufficient sleep time or decreased sleep quality, making it difficult to meet the physiological needs of patients and significantly affecting their daytime activities. Perimenopausal insomnia (PI) is a common perimenopausal syndrome in women, with an incidence of approximately 50–55%, which is significantly higher than the incidence of insomnia in other age groups [1]. Hot flashes are one of the main causes of PI and are reported to affect 80% of perimenopausal women [2]. Methods to treat PI are mainly divided into psychotherapy and hormone replacement therapy, but because of the inevitable side effects, their clinical application is limited [3]. In recent years, traditional Chinese medicine (TCM) is favored by an increasing number of patients because of its good efficacy and low side effects. Hot flushes are mostly caused by Yin deficiency and fire exuberance syndrome according to TCM, and Tianwang Buxin is the classical prescription for treating Yin deficiency and fire-flourishing insomnia. TWBXG is composed of semen platycladi, semen ziziphi spinosae, Asparaginase, Radix Ophiopogonis, dried radix rehmanniae, Angelica sinensis, ginseng, Radix scrophulariae, Salvia miltiorrhiza, Radix platycodi, Poria cocos, Polygala amflra, and Fructus schizandrae and mainly used for the treatment of insomnia, coronary heart disease, neurasthenia, etc. Our previous meta-analysis showed that Tianwang Buxin Dan can significantly improve the clinical treatment efficiency of insomnia patients [4], and the only difference between Tianwang Buxin Dan and Tianwang Buxin granule is that the dosage forms are different; the drug composition and indications of both are exactly the same. However, whether Tianwang Buxin granule (TWBXG) can treat PI has not been reported yet.

The human microbiome refers to the collective genomes of the microbes (composed of bacteria, bacteriophage, fungi, protozoa, and viruses) that live inside and on the human body [5]. Categories of human genes include the human genome and human microbiome, and the human symbiotic microbiome is considered as the “second human genome.” Microorganisms have been identified as the cause of mutations in many diseases and can affect host phenotype [6]. Recent studies have found that abnormalities in the intestinal flora are closely related to metabolic diseases such as type 2 diabetes. Intestinal flora helps maintain host circadian rhythms as well as host metabolome rhythms. Numerous epidemiological studies have shown that sleep deprivation is closely related to obesity and metabolism, and abnormalities in the intestinal flora are closely related to metabolic and neurological diseases [7–14]. Whether the occurrence and development of insomnia are also related to the abnormalities in intestinal flora has become a research hotspot in this field.

In this study, we observed 13 perimenopausal women with insomnia as the model group and their spouses as the control group to evaluate the relationship between PI and intestinal flora composition. PI patients were treated with TWBXG for 4 weeks to further explore whether TWBXG plays its therapeutic role by regulating the intestinal flora composition in PI patients.

## 2. Material and Methods

### 2.1. Ethical Approval

This study was conducted according to the guidelines laid down in the declaration of Helsinki, and all procedures involving human subjects were approved by the Ethics Committee of Hubei Province Hospital of TCM (SL2017-C04). All subjects were given oral and written information about the purpose and procedures of the study. Each participant signed an informed consent before the experiment, and the subjects were free to withdraw from the study at any time without any explanation. All experiments were registered in the Chinese clinical trial registry and approved by the World Health Organization clinical trial registration platform (ChiCTR-IPR-17011549).

### 2.2. Participant Information

Patients were enrolled from the Hubei Provincial Hospital of TCM, Hubei University of TCM, and Wuhan Traditional Chinese Medicine Hospital from March to October 2019. According to the inclusion and exclusion criteria, 120 cases were collected, consisting of 60 insomnia patients and their spouses. Among 15 female insomnia patients whose ages were within the perimenopause range (45–55 years old), two could not continue the follow-up consultation and automatically withdrew from the study. The inclusion criteria were as follows: (1) patients with insomnia as the main complaint and meeting DSM-V diagnostic criteria; (2) age: 40–65; (3) the spouse of the patient was not experiencing insomnia; (4) no special dietary habits or use of antibiotics, antiviral, or antifungal drugs in the past six months; (5) willing to provide blood and fecal specimens; and (6) signed informed consent. The exclusion criteria were as follows: (1) suffering from severe gastrointestinal diseases that have not been controlled and have undergone gastrointestinal surgery in the past 5 years; (2) diagnosed with inflammatory bowel disease, including ulcerative colitis, Crohn's disease, or indeterminate colitis; (3) persistent, infectious gastroenteritis, colitis, or gastritis; persistent or chronic diarrhea with unknown etiology, Clostridium difficile infection (relapse), or H. pylori infection (untreated); (4) irritable bowel syndrome; (5) chronic constipation; (6) suffering from severe diseases such as malignant tumors, cerebral infarction, and metabolic diseases; (7) patients with severe anxiety and depression; (8) pregnant and nursing patients; and (9) did not sign informed consent and did not provide blood, nails, or fecal specimens.

### 2.3. Drug Intervention Process

TWBXG was provided by Hubei Jinpai Liability Co., Ltd. All members in the group were given a Pittsburgh Sleep Index Scale (PSQI) score on the first day of the experiment. TWBXG was administered on the second day (half a bag, 83 g/bag, once in the morning and once in the evening), and the intervention continued for 4 weeks. The spouse of the patient was regarded as a control and was given a normal diet without additional drug intervention. On the 29th day of the experiment, the PSQI score was evaluated again to observe the effect of TWBXG on insomnia in perimenopausal women.

Clinical effectiveness was calculated based on the PSQI score: (1) clinical healing: sleep time returned to normal, deep sleep, full of energy after waking, accompanied by disappearance of major clinical symptoms, PSQI reduction rate ≥ 75%; (2) significant effect: sleep improved significantly, with most of the main clinical symptoms disappearing, 50% ≤ reduction rate < 75%; (3) effective: prolonged sleep time, with improvement of main clinical symptoms, 25% ≤ reduction rate < 50%; and (4) ineffective: no improvement in sleep quality, no significant improvement before and after symptomatic treatment, score reduction rate < 25%. Among all cases, those that were cured, markedly effective, and effective were judged to be effective. PSQI reduction rate = (total score before treatment − total score after treatment)/total score before treatment × 100%.

### 2.4. Stool Sample Collection

Stool samples were collected from all patients at home using the Longseegen Stool Storage Kit (FSCC-03, Longsee, China). The participants were told to collect specimens in advance, and the specimen should come from the end of the stool (the last 15% of every stool) and be free of urine and other possible pollutants (like blood or semen). After specimens were collected, they were placed in a prepared centrifuge tube immediately and stored in liquid nitrogen until further processing. DNA (approximately 0.18–0.2 g) was extracted from the stool samples in accordance with the instructions of the QIAamp DNA Stool Mini Kit (QIAGEN, Dusseldorf, Germany). The DNA concentration and quality were determined using a NanoDrop 1000 spectrophotometer (Thermo Scientific, MA, USA).

### 2.5. Determination of Intestinal Flora

After completing the NanoDrop to examine the purity of DNA, we randomly broke the qualified DNA samples into fragments with a length of about 350 bp using an ultrasonic crusher. The fragments were then subjected to end repair, 3′ end plus A, sequencing adapter, purification, fragment selection, PCR amplification, and other steps to complete the entire library preparation. After the library construction was completed, preliminary quantification was performed using electrophoresis and NanoDrop. Qubit was quantified for libraries with a concentration ≥ 15 ng/*μ*l, and the size of the insert fragments of the library was detected by capillary electrophoresis. After the insert size was as expected, the library was analyzed by qPCR quantitative accurate concentration (effective library concentration > 3 nM) to ensure library quality. The different libraries were mixed for Illumina sequencing according to the requirements of effective concentration and target offline data volume. KneadData software was used for quality control of the original data (Trimmomatic parameters: ILLUMINACLIP: adapters_path: 2:30:10 SLIDINGWINDOW: 4:20 MINLEN: 50) and dehosting (bowtie2 parameters: very sensitive); before KneadData and after KneadData, FastQC was used to check the rationality and effect of quality control [15, 16]. Kraken2 and other databases were used to identify the species contained in the sample. Bracken was used to predict the actual relative abundance of the species in the sample [17–20]. HUMAnN2 software (based on Diamond) was used to obtain annotation information and relative abundance table [21–24] of each functional database, and abundance clustering analysis was performed based on the species abundance tables and functional abundance tables [24, 25].

### 2.6. Statistical Analysis

Data are expressed as the mean ± standard deviation. To analyze the differences between groups, data comparison was performed by *t*-tests and one-way analysis of variance using SPSS 22 statistical software. *P* < 0.05 was considered statistically significant.

## 3. Results

### 3.1. General Condition

In this experiment, we included 13 PI patients (model group) and 13 healthy spouses (control group). We conducted statistics on the basic information of the members. There was no significant difference in the age, weight, and BMI between the two groups (*P* > 0.05) (Table 1).

### 3.2. PI Is Related to Intestinal Flora Disorders

**Figure 1:**
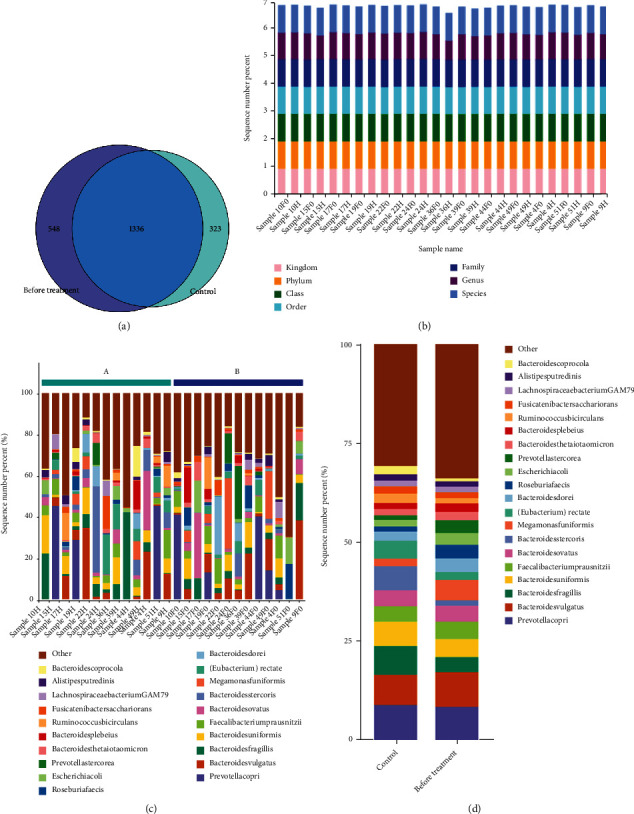
Metagenomic sequencing was performed to detect the composition changes in intestinal flora in the control group and before treatment group. (a) Venn diagram of species in control and before treatment groups. (b) Microbial communities were examined based on phylum, class, order, family, genus, and species in the control and before treatment group. (c, d) Relative abundance of predominant bacteria at the species level. H: control group; F0: before treatment.

**Figure 2:**
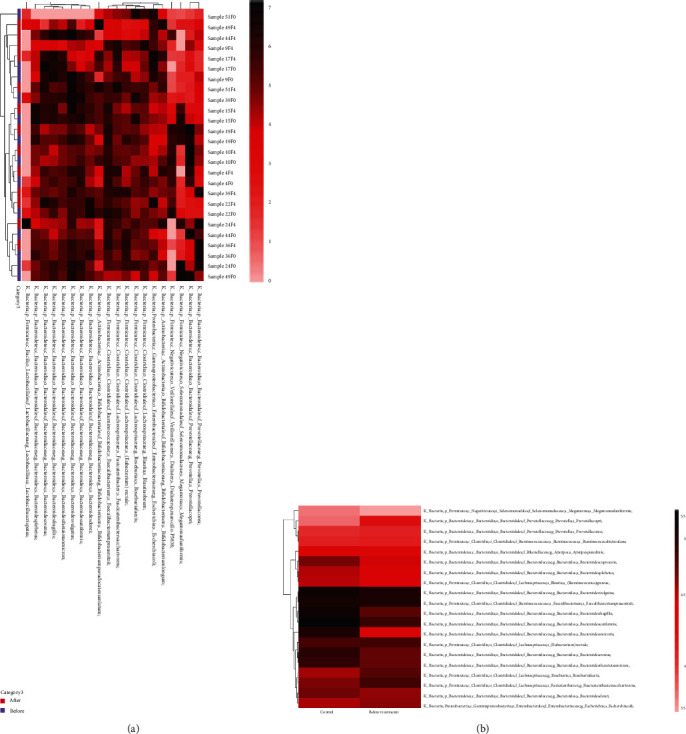
Heat map of common species in (a) all samples or (b) control and before treatment group. Darker colors represent higher abundance. The degree of similarity between samples and between microbiota is indicated by the dendrogram on the *x*- and *y*-axis, respectively.

We first scored the PSQI for the control and model groups. The average PSQI score of the model group was 15.38, which was much higher than that of the control group (*P* < 0.05). According to the PSQI standard, the model group was identified as an insomniac group. We then collected feces from the control and model groups and analyzed the composition of the intestinal flora. There were 1659 species in the control group and 1884 in the model group, and 1336 species were present in both groups (Figure 1(a)). We then analyzed the annotation degree among samples. The results showed that all samples stabilized the annotation at the 7 classification levels of kingdom, phylum, class, order, family, genus, and species, and the annotation degree among samples was similar (Figure 1(b)). At the species level, there were differences in the composition of Bacteroides, Prevotella copri, fecal Bacteroidetes, Macromonas monomorpha, Faecalibacterium prausnitzii, Roseburia faecis, and Ruminococcus between the two groups (Figures 1(c) and 1(d)). Further cluster analysis was performed on the samples to examine the compositional differences in intestinal flora between the two groups. Compared with the control group, the proportions of Blautia obeum, Roseburia faecis, Ruminococcus, Prevotella copri, and Fusicatenibacter saccharivorans in the model group increased, while those of Faecalibacterium prausnitzii, Bacteroides, and fecal Bacteroidetes decreased, implying that PI may be related to changes in the abundance of Blautia obeum, Roseburia faecis, Ruminococcus, Prevotella copri, Faecalibacterium prausnitzii, Bacteroides, fecal Bacteroidetes, and Fusicatenibacter saccharivorans (Figures 2(a) and 2(b)).

### 3.3. TWBXG Ameliorated Insomnia Symptoms in Perimenopausal Women

**Figure 3:**
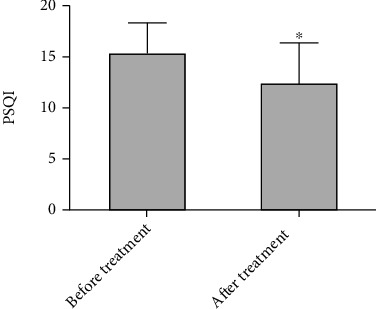
Effect of Tianwang Buxin granule on perimenopausal insomnia. ^∗^*P* < 0.05 vs. before treatment group.

To study the effect of TWBXG on insomnia in perimenopausal women, we assessed the PSQI scores of insomnia patients before and after treatment. The PSQI scores of patients after treatment were significantly reduced compared with those before treatment (*P* < 0.05), suggesting that TWBXG effectively ameliorated the symptoms of insomnia in perimenopausal women (Figure 3).

### 3.4. TWBXG Exerts Its Effect by Regulating the Composition of the Intestinal Flora

**Figure 4:**
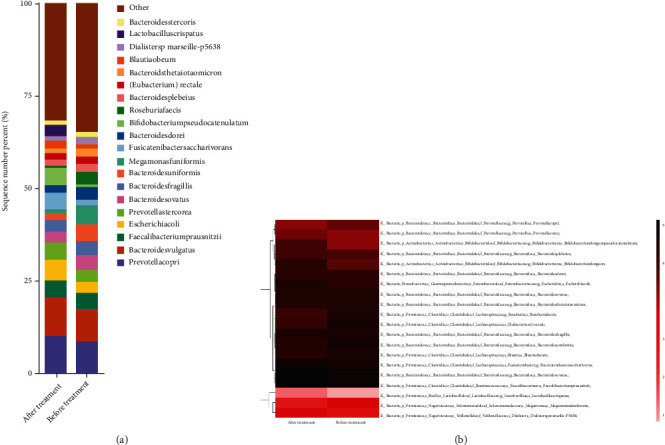
Composition of intestinal flora before and after treatment with Tianwang Buxin Granule. (a) Relative abundance of predominant bacteria at the species level. (b) Heat map of common species in the control and before treatment groups. Darker colors represent higher abundance. The degree of similarity between samples and between microbiota is indicated by the dendrogram on the *x*- and *y*-axis, respectively.

**Figure 5:**
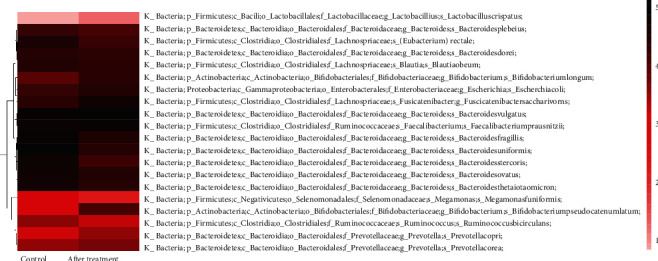
Heat map of common species in the control and after treatment groups. Darker colors represent higher abundance. The degree of similarity between samples and between microbiota is indicated by the dendrogram on the *x*- and *y*-axis, respectively.

In the foregoing experimental results, we confirmed that TWBXG effectively ameliorated the symptoms of insomnia in perimenopausal women. Whether TWBXG played its therapeutic role by altering the composition of the intestinal flora is still unclear. Therefore, in the following experiments, we further observed the intestinal flora composition of patients before and after treatment. Compared with pretreatment, the proportions of Blautia obeum, Roseburia faecis, Ruminococcus, Prevotella copri, and Fusicatenibacter saccharivorans were decreased, while those of Faecalibacterium prausnitzii, Bacteroides, fecal Bacteroidetes, Bifidobacterium, and Lactobacillus were increased after treatment (Figures 4(a) and 4(b)). As shown in Figure 5, the proportions of Blautia obeum, Roseburia faecis, Ruminococcus, Prevotella copri, Fusicatenibacter saccharivorans, Faecalibacterium prausnitzii, Bacteroides, and fecal Bacteroidetes were similar in the treatment group compared with those in the control group, suggesting that TWBXG alleviated the symptoms of insomnia by regulating the composition of intestinal flora in perimenopausal women.

## 4. Discussion

### 4.1. Principal Findings and Results

In the past 10 years, research on microorganisms has developed rapidly. Intestinal microorganisms not only have been reported to be related to metabolic diseases and cancer but are also closely related to nervous system diseases such as Parkinson's disease and ALS. A series of new research ideas such as the enterocerebral axis and the enterohepatic axis have been summarized.

Valles-Colomer et al. [26] conducted a large cohort study of 11.5% patients with depression and showed that regardless of whether patients with depression received antidepressant treatment, their quality of life scores were significantly lower than those of healthy people. By analyzing their gut microbiota, some microbiological characteristics associated with depression and poor quality of life were identified in depressed patients, such as high-abundance Flavobacterium bacteria and low-abundance Coprobacter and fecal cocci bacteria. After controlling the use of antidepressant drugs, the composition of intestinal flora of depressed patients was still different from that of healthy people, and their microbacilli and fecal bacteria were significantly reduced compared with those of healthy people. Han et al. [27] used C. elegans as a model to screen nearly 4000 E. coli with different mutations in their bodies and found that dozens of them prolonged lifespan, delayed tumor development and *β*-amyloid accumulation, and combined intestinal microorganism-gene-aging-age-related diseases for the first time, providing a new idea for the practical application of research. Previous studies have shown that there is a close relationship between intestinal flora and neuropsychiatric diseases, and many diseases with unclear pathogenesis can find hints or even answers in the field of intestinal flora. Therefore, we speculate that there is an association between insomnia and abnormal intestinal flora in perimenopausal women.

Based on the hypothesis that PI is related to abnormal intestinal flora, we collected the feces of women with PI and their noninsomniac partners. We examined the composition and abundance of the intestinal flora by metagenomic sequencing to further verify our conjecture. To avoid experimental error caused by crowd gathering, we collected participants from three medical institutions. Disorder of the biological clock is closely related to diseases such as coronary heart disease, depression, Alzheimer's disease, diabetes, and hypertension. Different living habits can cause abnormalities in the body's biological clock, which can lead to diseases. Hence, we used couple control by collecting noninsomniac spouses of PI women as a control group to avoid the influence of diet and different biological clocks. Statistical analysis was performed on all samples in the control and model group. We observed that there were abnormal compositions of Roseburia faecis, Blautia obeum, Ruminococcus, Prevotella copri, Fusicatenibacter saccharivorans, Faecalibacterium prausnitzii, Bacteroides, and fecal Bacteroidetes between the control and model groups. Previous studies showed that the abnormality of flora was closely related to numerous diseases. Wang et al. found that abnormal content of Ruminococcus, Blautia obeum, Faecalibacterium prausnitzii, etc. is closely related to coronary heart disease [28]. Gao et al. found that the proportion of Blautia obeum, Faecalibacterium, Prevotella copri, Roseburia faecis, and Ruminococcus in the intestine of patients with colon cancer decreased in varying degrees (*P* < 0.05) [29]. Suhana et al. tested the skin swabs and stool samples of 13 patients and found that lichen sclerosus may be associated with abnormal levels of Roseburia faecis and Ruminococcus [30]. We further studied whether the effect of TWBXG was achieved by regulating the disordered intestinal flora by treating 13 insomniac patients with TWBXG for 4 weeks. After treatment, the proportions of Roseburia faecis, Blautia obeum, Ruminococcus, Prevotella copri, and Fusicatenibacter saccharivorans were decreased, while those of Faecalibacterium prausnitzii, Bacteroides, fecal Bacteroidetes, Bifidobacterium, and lactobacillus were increased. However, compared with the control group, there was a certain gap in the abundance of the related flora in the treated group. This is also consistent with the efficacy of TWBXG in treating insomnia in this trial. Before and after TWBXG treatment, we observed a significant increase in the abundance of Bifidobacteria and Lactobacillus, suggesting that TWBXG not only restored the disordered flora to a normal state but also mobilized probiotics to achieve a synergistic effect.

The effect of TCM on intestinal flora is mainly exerted through small molecule metabolism, carbohydrate metabolism, targeting effect, and intestinal bacteria participating in the interaction between multiple components of Chinese herbal medicine [31]. TWBXG is composed of semen platycladi, semen ziziphi spinosae, Asparaginase, Radix Ophiopogonis, dried radix rehmanniae, Angelica sinensis, ginseng, Radix scrophulariae, Salvia miltiorrhiza, Radix platycodi, Poria cocos, Polygala amflra, and Fructus schizandrae. Previous studies have confirmed that ginseng promoted the increase in Lactobacillus and Bifidobacteria, improved the intestinal microbial composition, and thereby reduced the symptoms of ulcerative colitis [32]. Poria polysaccharide [33] and tanshinol [34] can also protect the human body by increasing the content of AKK, Bifidobacteria, and Lactobacilli and promoting the production of short-chain fatty acids [35].

### 4.2. Clinical Implications

This study provides a new idea for clinical treatment of perimenopausal insomnia, but due to the sample size of this study, more large-sample multicenter research results are needed to support it before it can be used in clinical treatment.

### 4.3. Strengths and Limitations

PI is a condition that most women will encounter during perimenopause; however, due to insufficient attention, there are few researches on PI at present. Soares et al. observed that eszopiclone can improve the sleep of perimenopausal women and have a positive impact on the mood, quality of life, and menopause-related symptoms of perimenopausal and early postmenopausal insomnia women [36]. The possible mechanism of action of eszopiclone was not further explored in Soares et al.'s study, leading to its limited clinical translational significance. In this study, based on the observation of the effect of TWBXG on PI, we further discussed its potential mechanism and increased the possibility of its clinical transformation. In this study, we found that after TWBXG intervention, the intestinal flora disorder of perimenopausal insomniac patients was relieved. However, there is still a certain gap in the abundance of the varied flora compared with that in normal people. The regulation of disordered intestinal flora and the recolonization of the intestinal flora are a long-term process. Most patients in this study had severe insomnia and relatively short courses of treatment, therefore leading to the incomplete recovery of the disordered flora after TWBXG intervention in this trial. We will extend the intervention experiment in subsequent studies to further confirm the effect of TWBXG on the intestinal flora of patients with PI. In this study, we only observed the effect of TWBXG on the composition and abundance of intestinal flora in PI patients, but how TWBXG affects the function of the flora and its specific mechanism of action has not been observed. In future experiments, we will study the effects of TWBXG on the functional metabolism and potential mechanisms of different strains based on further data mining.

## 5. Conclusion

Our study demonstrated that PI is associated with intestinal flora disturbance, which is mainly manifested as an increase in the abundance of Roseburia faecis, Ruminococcus, Prevotella copri, Fusicatenibacter saccharivorans, and Blautia obeum and a decrease in the abundance of Bacteroides, fecal Bacteroidetes, and Faecalibacterium prausnitzii. TWBXG can be used to effectively treat PI, and its effect may be achieved by regulating the disordered intestinal flora in PI patients.

## Figures and Tables

**Table 1:** General condition (*n*, *x* ± *s*).

Group	*n*	Age	Weight	BMI
Control	13	50.28 ± 6.84	61.39 ± 10.00	23.23 ± 2.71
Model	13	50.19 ± 6.25	59.74 ± 9.66	22.73 ± 2.71

## Data Availability

The data sets generated for this study are available on request to the corresponding author.

## Additional Points

*Statement of Significance*. Currently, there is no ideal method for the treatment of perimenopausal insomnia. Tianwang Buxin granule has been used in the clinical treatment of perimenopausal insomnia in traditional Chinese medicine for many years, and its curative effect is relatively stable, but its mechanism is not clear, which seriously restricts its popularization and application. The disorder of intestinal flora has been proved to be closely related to many human diseases; thus, we wondered if perimenopausal insomnia might also be associated with intestinal microbiome disorders, and based on this hypothesis, we performed relevant tests, and the results showed that perimenopausal insomnia is related with intestinal flora disorders and Tianwang Buxin granules can effectively relieve the symptoms of perimenopausal insomnia. Our study provides a new idea and potential treatment for perimenopausal insomnia.
